# Opsin3 Downregulation Induces Apoptosis of Human Epidermal Melanocytes *via *Mitochondrial Pathway

**DOI:** 10.1111/php.13178

**Published:** 2020-01-10

**Authors:** Yu Wang, Yinghua Lan, Hongguang Lu

**Affiliations:** ^1^ Department of Dermatology Affiliated Hospital of Guizhou Medical University Guiyang Guizhou China

## Abstract

G protein‐coupled receptors (GPCRs) are core switches connecting excellular survival or death signals with cellular signaling pathways in a context‐dependent manner. Opsin 3 (OPN3) belongs to the GPCR superfamily. However, whether OPN3 can control the survival or death of human melanocytes is not known. Here, we try to investigate the inherent function of OPN3 on the survival of melanocytes. Our results demonstrate that OPN3 knockdown by RNAi‐OPN3 in human epidermal melanocytes leads to cell apoptosis. The downregulation of OPN3 markedly reduces intracellular calcium levels and decreases phosphorylation of BAD. Attenuated BAD phosphorylation and elevated BAD protein level alter mitochondria membrane permeability, which trigger activation of BAX and inhibition of BCL‐2 and raf‐1. Activated BAX results in the release of cytochrome c and the loss of mitochondrial membrane potential. Cytochrome c complexes associate with caspase 9, forming a postmitochondrial apoptosome that activate effector caspases including caspase 3 and caspase 7. The release of apoptotic molecules eventually promotes the occurrence of apoptosis. In conclusion, we hereby are the first to prove that OPN3 is a key signal responsible for cell survival through a calcium‐dependent G protein‐coupled signaling and mitochondrial pathway.

## Graphical abstract

Opsin3 (OPN3) is a key signal responsible for cell survival through a calcium‐dependent G protein‐coupled signaling and mitochondrial pathway. Our research shows that OPN3 knockdown by RNAi‐OPN3 in human epidermal melanocytes leads to apoptosis. Furthermore, the knockdown of OPN3 in human epidermal melanocytes results in a decrease in intracellular calcium levels and then activation of downstream cell signaling pathway.

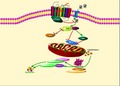

## Introduction

Melanocytes are pigment‐producing cells of the skin in humans and other vertebrates 1 whose number and function determine the skin color. The survival, proliferation, migration and self‐renewal of melanocytes are regulated by internal and external environmental factors. Several protein molecules, which regulate melanocyte survival including microphthalmia transcription factor (MITF), c‐kit, snail/slug, sox10 and endothelins 2, 3, 4, 5, have been identified. However, the key switch molecules that control survival or apoptosis signals of melanocytes have not been identified.

Recently, it has been suggested that cell survival may be associated with G protein‐coupled receptors (GPCRs) 6, 7. GPCRs can promote cell survival of melanocytes through acting with the extracellular sphingosine 1‐phosphate (S1P) 6. Opsins (OPNs) belong to the GPCR superfamily 8, 9. However, whether OPNs can control the survival or death of human melanocytes is not known.

Previous studies have demonstrated that OPNs play a pivotal role in non–image‐forming responses to light including physiological adaptations (of pupil size, circadian rhythm and activity) to ambient light 10, 11, 12, 13. In recent years, multiple light‐independent roles of OPNs have been found including developmental, visual, affective and cognitive functions 14, 15, 16, 17. Abnormal expression of OPNs may cause apoptosis of photoreceptor cells in the retina 18, which is thought to be due to altered intrinsic functions (gene mutation) of OPNs.

Opsin 3 (OPN3) (encephalopsin, panopsin), a nonvisual optic protein, is mainly expressed in the eye, skin, brain, liver and kidney 11, 19, 20, 21, 22. Interestingly, OPN3 has light‐independent roles in the asthma and cell cycle modulation of hair follicle cells 23. OPN3 has also recently been found in human epidermal melanocytes 12, 24, 25, but the inherent functions of OPN3 remain to be elucidated.

Here, we report that OPN3 is a key molecule responsible for survival of human epidermal melanocytes. Knockdown of OPN3 in human epidermal melanocytes results in a decrease in intracellular calcium levels and activation of the downstream cell signaling pathway. Downregulation of OPN3 markedly reduces the intracellular calcium level and decreases the phosphorylation level of BAD. The reduced amount of phosphorylated BAD and elevated level of BAD alter mitochondria membrane permeability, which trigger activation of BAX and inhibition of BCL‐2 and raf‐1, leading to the conventional apoptosis pathway. In conclusion, we hereby are the first to prove that OPN3 is a key signal responsible for cell survival through a calcium‐dependent G protein‐coupled signaling and mitochondrial pathway.

## Materials and Methods

### Cell culture

Normal human epidermal melanocytes (NHMs) were obtained from child foreskin with two‐step enzyme‐digestion method as described previously elsewhere. Cells were cultured in Medium 254 (Gibco, M254500) containing human melanocyte growth supplements (HMGS2; Gibco, S0165), 2 mm L‐glutamine (Gibco, 1051024) and penicillin–streptomycin (Solarbio, China, P1400). Cells were cultured at 37°C in a humidified incubator (Forma) with 5% CO_2_ and used at their third passage.

### qRT‐PCR assay

Total RNA was isolated from cultured NHMs using TRIzol (Invitrogen, 15596026), and reverse transcription was performed from 0.3 μg of total RNA using RevertAid RT Reverse Transcription Kit (Invitrogen, K1691) according to the manufacturer's instructions. qRT‐PCR was performed using a Mastercycler ep realplex real‐time PCR system (Eppendorf, German) with SYBR Green PCR Master Mix (TIANGEN, Beijing, China, FP402) in the amplification reaction mixtures (25 μL). Relative opsin RNA expression was calculated using the 2^‐ΔCt^ method, and human GAPDH was used as an internal control. All reactions were performed as triplicates. The following human primers were used in this study.

OPN1‐SW; forward (fwd)5′‐TGTGCCTCTCTCCCTCATCT‐3′,
reverse (rev) 5′‐GGCACGTAGCAGACACAGAA‐3′;
OPN2; forward (fwd)5′‐GAGTCAGCCACCACACAGAA‐3′,
reverse (rev) 5′‐CATGAAGATGGGACCGAAGTTGGAG‐3′;
OPN3; forward(fwd) 5′‐CAATCCAGTGATTTATGTCTTCATGATCAGAAAG‐3′,
reverse (rev) 5′‐GCATTTCACTTCCAGCTGCTGGTAGGT‐3′;
OPN4; forward (fwd)5′‐TCCTCCTCTTCGTGCTCT‐3′,
reverse (rev) 5′‐GTAAATGATGGGGTTGTGG ‐3′;
OPN5; forward (fwd)5′‐CTAGACGAAAGAAGAAGCTGAGACC‐3′,
reverse (rev) 5′‐GCGGTGACAAAAGCAAGAGA‐3′;
GAPDH; forward (fwd) 5′‐GACATCCGCAAAGACCTG‐3′,
reverse (rev) 5′‐GGAAGGTGGACAGCGAG ‐3′.

### Western blot assay

Protein extracts were obtained by cell lysis in RIPA lysis buffer (Solarbio, Beijing, China, R0010) containing 1mM PMSF (Solarbio, R0010). 40 μg of proteins was subjected to SDS‐PAGE and then transferred onto polyvinylidene difluoride (PVDF) membranes. The membranes were blocked with nonfat milk overnight at 4°C. After washing three times with Tris‐buffered saline Tween washing buffer (TBST buffer), the membranes were incubated with primary antibodies for 2 h at room temperature. Then, the membranes were washed again for four times and incubated with HRP‐conjugated goat anti‐mouse IgG affinity‐purified antibody (1:2000, MDL, Beijing, China, MD912524) or HRP‐conjugated goat anti‐rat IgG affinity‐purified antibody (1:2000; MDL, MD912526) for 45 min at room temperature. Specific bands were visualized using ECL Western blot detection reagent (7sea Biotechnology, Shanghai, China, E003‐050) after washing six times with TBST buffer. The protein expression levels were measured by FastStone Image Viewer 5.5 software after normalization with β‐actin. Rabbit monoclonal antihuman OPN1 (1:1000; MDL, MDL11128), rabbit monoclonal antihuman OPN2 (1:1000; MDL, MDL11129‐100), rabbit monoclonal antihuman OPN3 (1:1000; MDL, MD4034), rabbit monoclonal antihuman OPN4 (1:1000; MDL, MD4194), rabbit monoclonal antihuman OPN5 (1:1000; MDL, MD4195), rabbit monoclonal antihuman caspase 3 (1:1000; MDL, MD6512‐100), rabbit monoclonal antihuman cleaved caspase 3 (1:1000; Cell Signaling Technology, Danvers, #9661T), rabbit monoclonal antihuman caspase 7 (1:1000; MDLMD4444‐50), rabbit monoclonal antihuman cleaved caspase 7 (1:1000; Cell Signaling Technology, #8438T), rabbit monoclonal antihuman caspase 9 (1:1000, MDL, MD1789‐50), rabbit monoclonal antihuman cleaved caspase 9 (1:1000; Abcam, Cambridge, UK, ab2324), rabbit monoclonal antihuman BAD (1:1000; MDL, MD5306‐50), rabbit monoclonal antihuman phospho‐BAD (1:1000; MDL, MD1656‐50), rabbit monoclonal antihuman BAX (1:1000; MDL, MD45‐100), rabbit monoclonal antihuman raf‐1 (1:1000; MDL, MD730‐50), rabbit monoclonal antihuman cytochrome c (1:1000; MDL, MD69‐50) and rabbit monoclonal antihuman bcl‐2 (1:1000; MDL, MD4835‐100) were used as primary antibodies. Mouse monoclonal antihuman β‐actin (1:1000; MDL, BS409‐100) was used as an internal control. As secondary antibodies, the blots were probed with horseradish peroxidase‐labeled IgG goat anti‐rabbit antibodies (1:2000; MDL, BS912565) or horseradish peroxidase‐labeled IgG goat anti‐mouse (1:2000; MDL, MD912524).

### Immunofluorescence assay

NHMs were inoculated on cell climbing sheets at a density of 10^4^ cells at 37°C with 5% CO_2_ for 24 h. After being washed three times with 0.1M PBS buffer solution (Solarbio, Beijing, China, P1010‐2L), cells were fixed with 95% ethanol at room temperature for 15 min and dried at room temperature. The cells were then blocked with bovine serum for 30 min at 37°C and washed three times with 0.1 m PBS buffer solution again. Following incubation with the prime antibody (mouse monoclonal antihuman OPN3, 1:50; MDL, MD6636‐100) at 4°C overnight, the cells were washed with 0.1 m PBS buffer solution for 5 min three times and covered with goat anti‐mouse IgG FITC‐labeled fluorescent antibody (1:50; MDL, MD6640‐100) for 1 h at 37°C, finally stained by 40,6‐diamidino‐2‐phenylindole (DAPI) (Gibco, D21490). The expression of OPN3 in NHMs was visualized under the confocal microscope (ZEISS, German). NHMs were also double labeled with mouse monoclonal antihuman OPN3 (1:50; MDL, MD6636‐100) and rabbit monoclonal antihuman melan‐A (1:50; Bioss, Beijing, China, bs‐7362R). The immunohistochemistry protocol used was described above, and cells were visualized under Cell Observer‐Living Cells (Zeiss).

### Generation of OPN3 knockdown NHMs

Knockdown of OPN3 in NHMs was performed using siRNA technology according to the manufacturer's protocol. Three pooled siRNA oligos targeting OPN3 or negative control siRNAs were purchased from ViewSolid Biotech (Beijing, China). The silence efficiency of above three different siRNA sequences was analyzed 48 h post‐transfection *via* quantitative RT‐PCR compared with negative control siRNAs not targeting any known gene. The siRNA sequence having the strongest silence efficiency on OPN3 was used for the follow‐up study. Levels of OPN3 gene silencing were assessed 24, 48 and 72 h post‐transfection by qPCR, respectively. 48 h was selected to be the time‐point for observation in later experiment. Controls included no siRNA transfection and negative control siRNA. The third passage NHMs were seeded in 6‐well plates at a concentration of 10^4^ cell/well. When reaching 60% confluence, cells were transfected using Lipofectamine 2000 (View Solid Biotech, Beijing, China) with a final siRNA concentration of 30 nm. After siRNA transfection, cells were cultured for 48 h for further detection. Levels of OPN3 gene and protein silencing were assessed 48 h post‐transfection by qPCR and Western blotting. The OPN3 siRNA sequences were as follows: 5′‐ACCUCCUC CUGGUCAACAUTT‐3′, 5′‐GUCACCUUUACCUUCGUGUTT‐3′, 5′‐CAAUUCAAGUGAUCAAGAUTT‐3′ and 5′‐UUCUCCGAACGUGUCACGUTT‐3′ as the sequence of the control. The OPN3 siRNA sequences (5′‐GUCACCUUUACCUUCGUGUTT‐3′) reduced the level of OPN3 mRNA by more than 80% compared with control siRNA sequence.

### Morphological analysis

The third passage NHMs were seeded onto cell climbing sheets in 6‐well plates at a concentration of 10^4^ cell/well for 24 h. Then, cells were transfected using Lipofectamine 2000 (View Solid Biotech) with a final RNAi‐OPN3 concentration of 30 nm. 48 h post‐transfection, cell morphological changes in NHMs were observed under Cell Observer‐Living Cells (Zeiss). In addition, cells on climbing sheets were also fixed in 95% ethanol and stained with DAPI (1.5 μg mL^−1^; Gibco, D21490). The photomicrographs of nuclear morphology were taken with Cell Observer‐Living Cells (Zeiss). Controls included no siRNA transfection and negative control siRNA.

### Mitochondrial membrane potential (MMP)

MMP of NHMs was measured using JC‐1 Dye (lipophilic cationic probe 5,5 V,6,6 V‐tetrachloro‐1,1 V,3,3 V‐tetraethylbenzimidazol carbocyanine iodide) (Molecular Probes, Solarbio, Beijing, China, M8650). Knockdown of OPN3 in the NHMs was performed using siRNA technology as described above. 48 h post‐transfection, NHMs were incubated with 2 mL M254 medium containing 5 mg mL^−1^ JC‐1 for 20 min at 37°C. Green fluorescence signals from intracellular JC‐1 dye indicate breakdown of MMP, that is mitochondrial damage. The signals were visualized using Cell Observer‐Living Cells (Zeiss). Control groups included no siRNA transfection and negative control siRNA.

### Flow cytometry analysis

The annexin V‐FITC/PI double staining was used to determine apoptotic cells on BD FACSCalibur Flow Cytometer (BD Biosciences, San Jose, CA). The third passage NHMs were seeded into 6‐well plates at a concentration of 10^4^ cell/well. When reaching 60% confluence, cells were transfected with a final siRNA concentration of 30 nm as described above. 48 h post‐transfection, cells were harvested and resuspended with 400 μL annexin buffer containing 5 μL of annexin V‐FITC (7sea Biotechnology, Shanghai, China, A005‐2). Cells were incubated for 15 min at room temperature in the dark conditions. After 10 μL of PI was added into above buffer, cells were incubated for 5 min in ice‐bath under the dark conditions. Cells were then detected by flow cytometry and analyzed using Cell Quest software. Controls included no siRNA transfection and negative control siRNA.

### Transmission electron microscopy

The third passage NHMs (60% confluency), transfected with a final siRNA concentration of 30 nm as described above for 48 h, were harvested and fixed overnight with 2.5% glutaraldehyde (pH 7.4) in 0.1 m PBS buffer solution (Solarbio, P1010‐2L) at 4°C. After being washed three times each for 10 min with 0.1 m PBS buffer solution, cells were fixed for 1 h with 1% osmium tetroxide acid at 4°C and rinsed three times each for 10 min with 0.1 m PBS buffer solution again. The dehydration was performed for 10 min per stage of a graduated series of acetone (50%, 70%, 80%, 90%) and 20 min with acetone (100%) for two times at room temperature. Then, samples were embedded in embedding medium (SPI‐PON812, SPI Supplies) diluted 1:1 with 100% acetone for 1 h, embedding medium (SPI‐PON 812, SPI Supplies) diluted 3:1 with 100% acetone for 1 h and embedding medium (SPI‐PON 812, SPI Supplies) for 1 h. The resin blocks were polymerized for 24 h at 45°C, followed by 48 h at 60°C and sectioned using Ultracut UCT Ultramicrotome (Leica, German) equipped to cut serial sections (average thickness 60 nm). Grids containing the sections were stained at room temperature using 2% (w/v) aqueous uranyl acetate for 10 min and Reynolds lead citrate for 3 min. All samples were analyzed using a HITACHI 7650 electron microscope (HITACHI Company, Japan), and digital acquisitions were made with a numeric camera (Keen View; Soft Imaging System, SIS, Germany). Controls included no siRNA transfection and negative control siRNA.

### Calcium imaging and image analysis

The third passage NHMs (60% confluency) were transfected for 48 h with a final siRNA concentration of 30 nm as described above. Cells were washed once with 0.1 m PBS buffer solution and incubated with 1 mL M254 medium containing 2.5 μm Fluo‐3/AM (MULTISCIENCES (LIANKE) BIOTECH, Hangzhou, China, F1243) for 30 min at 37°C in the darkroom. Fluorescent images of Fluo‐3/AM‐loaded cells were acquired using Cell Observer‐Living Cells (Zeiss, German). Controls included no siRNA transfection and negative control siRNA. In addition, cells were also harvested and incubated with 1 mL M254 medium containing 2.5 μm Fluo‐3/AM for 30 min at 37°C in the darkroom. Fluo‐3/AM‐loaded NHMs were centrifuged at 1000 g for 5 min at room temperature, washed one time with 0.1 m PBS buffer solution and resuspended with 0.5 mL 0.1 m PBS buffer solution. Then, the concentration of intracellular free calcium ion was measured with flow cytometric assay (BD Biosciences, San Jose, CA). The excitation source for Fluo‐3/AM was a 488‐nm air‐cooled argon laser and a 525‐nm band‐pass filter.

### Statistical analysis

Statistical analysis was performed with a statistic package (GraphPad Prism 7, GraphPad Software San Diego, CA). Data are represented as the mean ± SEM of three–four independent experiments. Differences between groups were assessed by one‐way analysis of variance (ANOVA) followed by Dunnett's or Bonferroni's test. *P* value < 0.05 was considered statistically significant.

## Results

### Overexpression of OPN3 on the plasma membrane of NHMs *in vitro*

**Figure 1:**
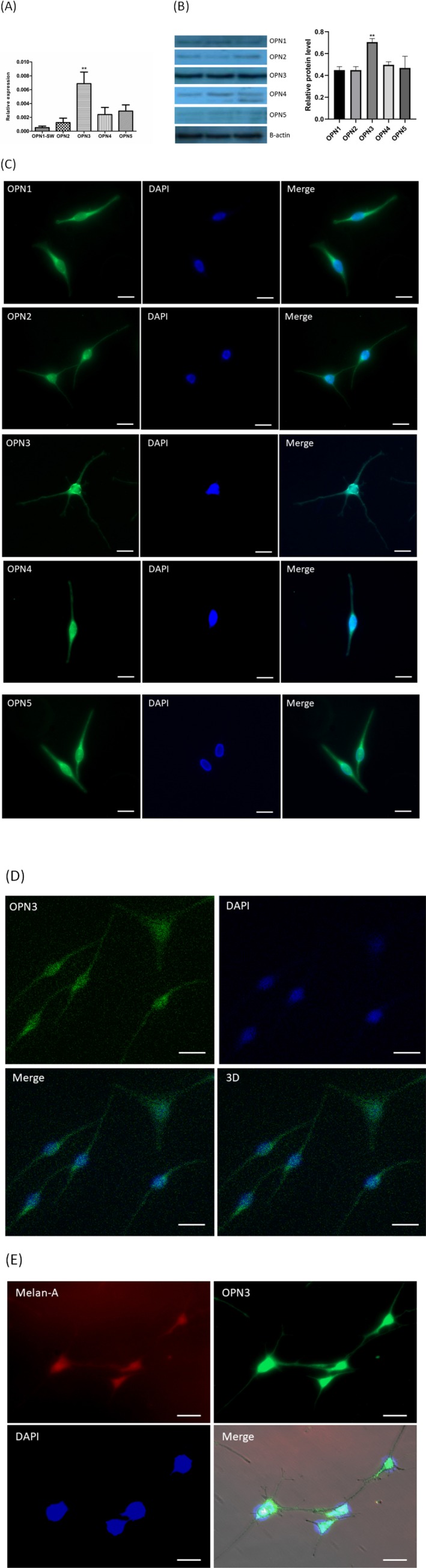
OPN expression in normal human epidermal melanocytes (NHMs). (A) Relative expression level of OPNs was analyzed by qPCR. OPN level was normalized with GAPDH level (*n* = 8). (B) The cell lysate was analyzed by Western blot using anti‐OPNs antibody and β‐actin. The relative protein level was quantified using Quantity One software (*n* = 3). (C) Immunofluorescence of normal human epidermal melanocyte with specific antibodies against OPNs (yellow), and nuclei (blue) under the fluorescence microscope. Scale bar = 20 μm. (D) Immunofluorescence of normal human epidermal melanocyte with specific antibodies against OPN3 (yellow), and nuclei (blue) under the confocal microscope. Scale bar = 20 μm. (E) OPN3 colocalized with melan‐A in human epidermal melanocytes with immunofluorescence double staining method. Photomicrographs were taken with Cell Observer‐Living Cells (Zeiss) and pseudocolored with Axiovision software (Zeiss). Scale bar = 20 μm (400 × magnification).

The expression of OPN subfamilies (OPN1, OPN2, OPN3, OPN4, OPN5) in mRNA and protein level of NHMs were firstly detected by qRT‐PCR and Western blot analysis. Our results are consistent with previous studies (Fig. 1A,B) 24, 26, and the expression of OPN3 is significantly higher than that of other OPNs (Fig. 1A,B). We further determined the subcellular localization of OPN protein in NHMs by confocal microscopy (Fig. 1C,D). We performed immunofluorescence double staining with antibodies against melanocyte‐specific protein melan‐A and OPN3. Positive melan‐A staining is a marker for identifying melanocytes 27. Melan‐A–positive NHMs showed a strong staining of OPN3 in the cell membrane (Fig. 1E).

### RNAi knockdown of OPN3 in NHMs leads to apoptosis

**Figure 2:**
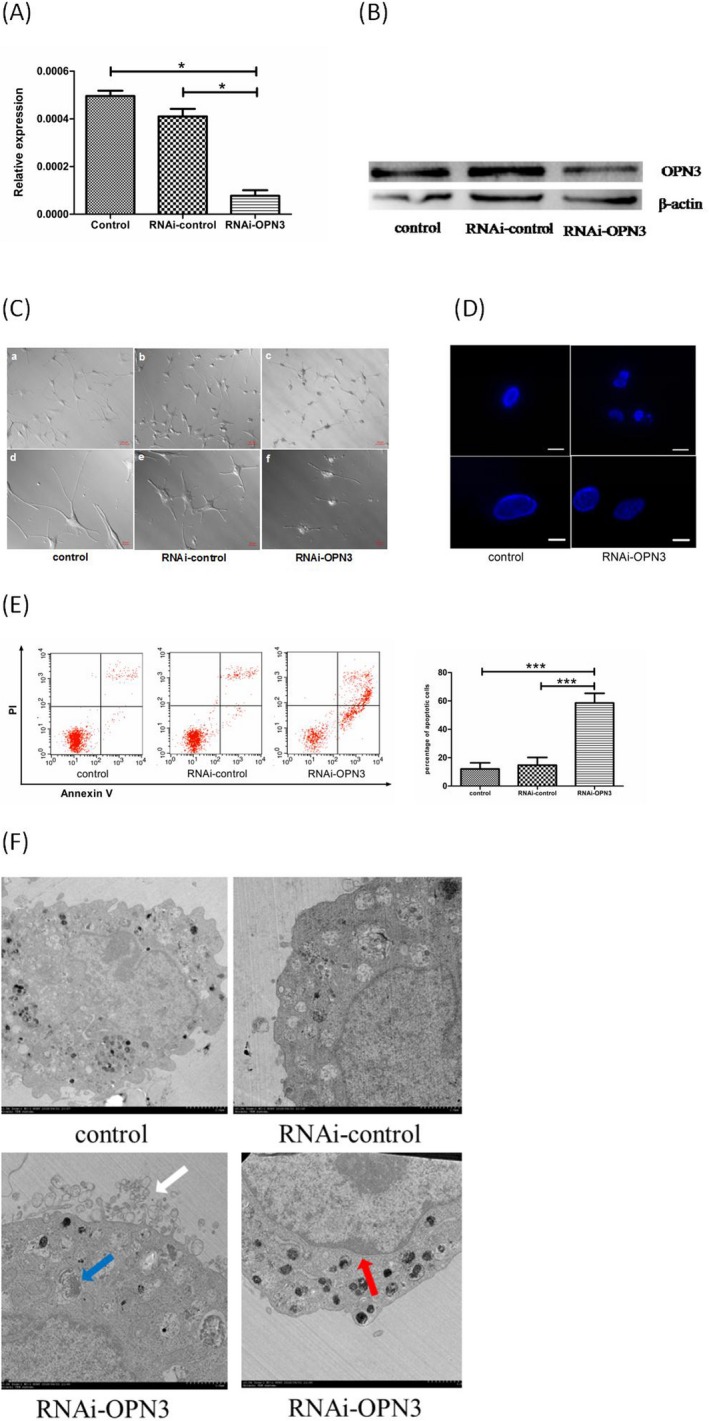
Apoptosis of human epidermal melanocytes following transfection with RNAi‐OPN3. (A) The expression of OPN3 decreased significantly at the mRNA level 48 h following transfection of NHMs with siRNA (*P* < 0.05). It was nearly unchanged in the melanocytes transfected with RNAi‐control and in the negative control group. The mRNA level of OPN3 was normalized with GAPDH levels (*n* = 3 independent experiments). *P < 0.05. (B) The expression of OPN3 also decreased significantly at the protein level compared with that in the RNAi‐control group and in the negative control group 48 h following transfection of NHMs with siRNA *in vitro* (*P* < 0.05). (C) The morphologic change after transfection with RNAi‐OPN3 was observed including cell fragmentation and cell shrinkage under the light microscopy. Little morphological changes occurred in the melanocytes transfected with RNAi‐control and in the negative control group. Photomicrographs were taken with Cell Observer‐Living Cells (Zeiss). Scale bar 50μm (200 × magnification). Scale bar 20 µm (400 × magnification). (D) The nuclear pyrosis and nuclear fragmentation were observed under fluorescent microscopy using DAPI staining after transfecting with RNAi‐OPN3. Photomicrographs were taken with Cell Observer‐Living Cells (Zeiss). Fine scale bar 50 μm (200 × magnification). Heavy scale bar 20 μm (400 × magnification). (E) Flow cytometry with annexin V‐FITC/ PI double staining was used to determine apoptotic cells. Following OPN3 knockdown, a significant increase in apoptosis was detected in the melanocytes transfected with RNAi‐OPN3 compared with controls. (F) Nuclear fragmentation (red arrow), budding (white arrow) and the formation of apoptotic bodies (blue arrow) were found under electron microscopes in the melanocytes transfected with RNAi‐OPN3.

We then knocked down OPN3 in NHMs using RNAi‐based gene silencing according to previous report 24. The expression of OPN3 decreased significantly at mRNA and protein level 48 h after transfection of NHMs with siRNA in vitro (Fig. 2A,B). Surprisingly, significant changes in morphology of melanocytes transfected with RNAi‐OPN3 were observed, including cell shrinkage and cell fragmentation under light microscopy (Fig. 2C) 28, 29. As expected, the cell morphology was nearly unchanged in the melanocytes transfected with RNAi‐control and in the negative control group (Fig. 2C). The nuclear pyrosis and nuclear fragmentation were observed under the fluorescent microscopy using DAPI staining in knocked down cells (Fig. 2D) 30. Those morphologic changes indicate cell death of NHMs after downregulating OPN3.

To further determine the mechanism of morphological changes, flow cytometry with annexin V‐FITC/PI double staining 31 was used to measure the survival rate of melanocytes 48 h after transfection of melanocytes with siRNA. 58.57 ± 5.56% of cells was observed to be apoptotic in the melanocytes transfected with RNAi‐OPN3, while only 14.67 ± 4.49% in the RNAi‐control transfecting group and 12.07 ± 3.54% in the control group (*P* < 0.01) (Fig. 2E). Moreover, nuclear fragmentation, budding and the formation of apoptotic bodies were also found under transmission electron microscopes (Fig. 2F) 30. These results indicate that cell and nuclear fragmentation in melanocytes transfected with RNAi‐OPN3 may be due to apoptosis.

### Inhibition of OPN3 promotes apoptosis of NHMs by calcium‐dependent G protein‐coupled signaling and mitochondrial pathway

**Figure 3:**
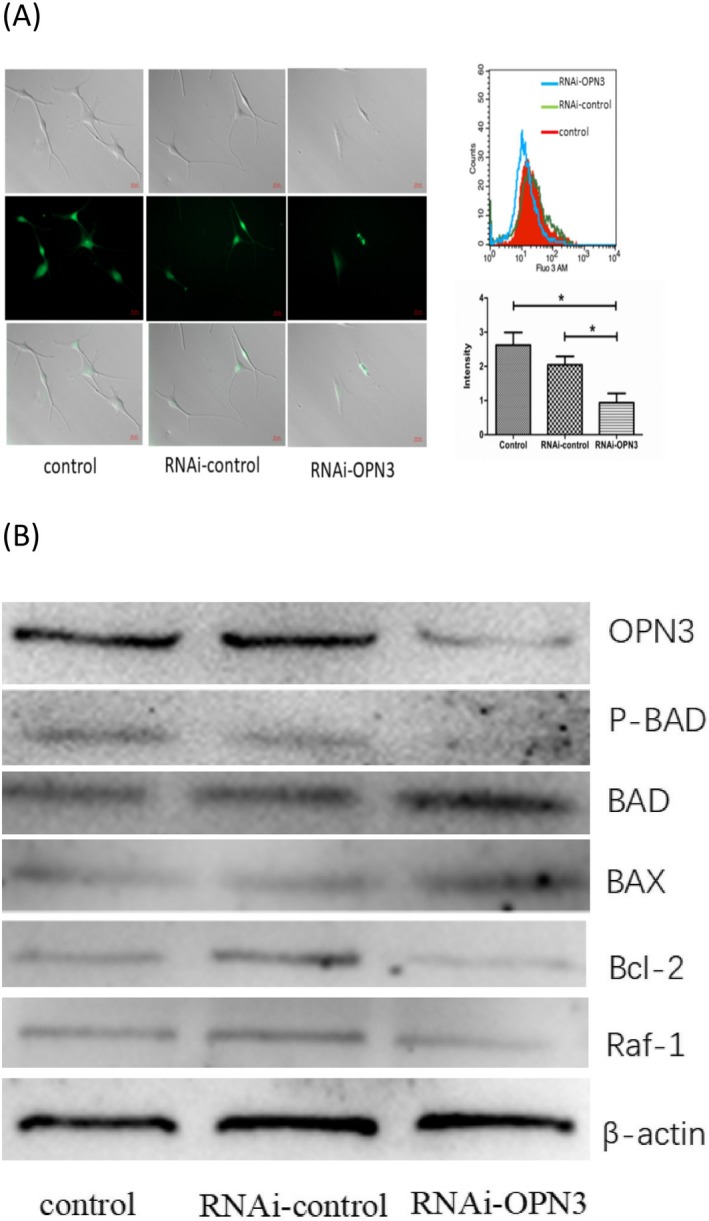
Intracellular Ca^2+^ reduction induced apoptosis through dephosphorylation of BAD following OPN3 knockdown in human epidermal melanocytes. (A) The reduced intracellular calcium level was detected through fluorometric Ca^2+^ imaging and flow cytometry following OPN3 knockdown in human epidermal melanocytes. (B) The reduced amount of phosphorylated BAD and elevation level of BAD triggered activation of BAX and inhibition of BCL‐2 and raf‐1.

Apoptosis plays an important role in the development and maintenance of tissue homeostasis 32, 33. How can OPN3 knockdown lead to apoptosis? Previous studies have suggested that phototransduction results in the conformational change in OPNs and promotes cGMP‐gated or transient receptor potential channels open 11, 12. To study the cellular and molecular mechanism through which OPN3 regulates apoptosis, we first investigated the calcium functions. After OPN3 knockdown in human epidermal melanocytes, the intracellular calcium level was decreased by fluorescence Ca^2+^ imaging. We further confirmed the intracellular calcium level reduction by flow cytometry (Fig. 3A). Decreased intracellular calcium levels result in dephosphorylation of BAD (Fig. 3B) 34. BAD is a proapoptotic member of the Bcl‐2 family that heterodimerizes with antiapoptotic proteins such as Bcl‐2 and Bcl‐xL, promoting cell death 34, 35. As previously reported, the reduction in phosphorylated BAD alters mitochondrial membrane permeability, triggering activation of BAX and inhibition of Bcl‐2 and raf‐1 36, 37, 38. In this study, we observed that Bcl‐2 and raf‐1 protein levels were significantly reduced in melanocytes after RNAi‐OPN3 transfection, and Bax protein expression levels were significantly increased (Fig. 3B).

**Figure 4:**
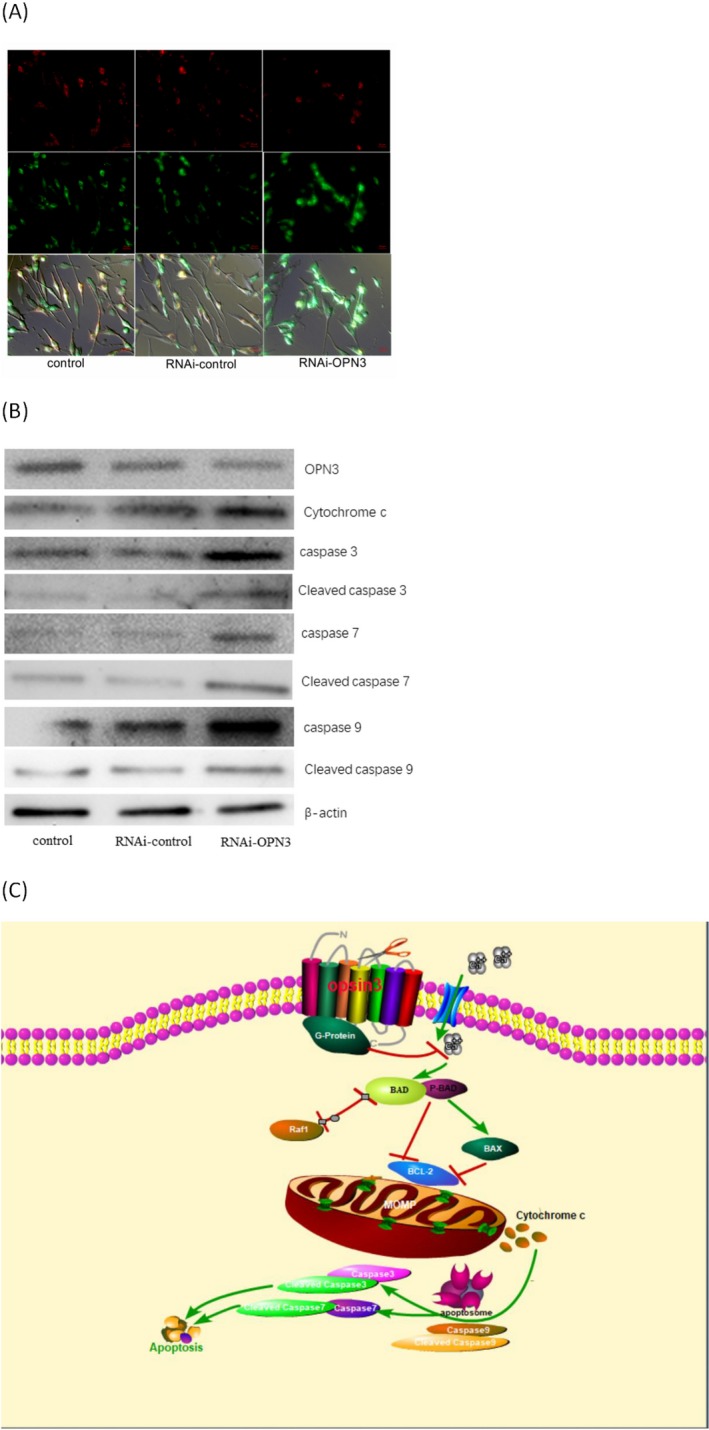
OPN3 knockdown induces apoptotic cell death through mitochondria pathway in human epidermal melanocytes. (A) Mitochondrial membrane potential (MMP) was reduced by OPN3 knockdown, which is shown by increased green cell fluorescence. Photomicrographs were taken with Cell Observer‐Living Cells (Zeiss) and pseudocolored with Axiovision software (Zeiss). Scale bar 20 μm (400 × magnification). (B) OPN3 knockdown upregulated the expression of apoptosis mitochondrial signal transduction pathway‐related proteins in human epidermal melanocytes. (C) The signal pathway map of our key finding in this study.

Existing studies have shown that lowering the Bcl2/Bax ratio promotes mitochondrial outer membrane permeabilization (MOMP) 39, 40, which is essential for apoptosis 41. Mitochondria transmembrane potential (MMP) decreases following MOMP during apoptosis 40, 42. We used JC‐1 staining method 43 to detect MMP of RNAi‐OPN3 melanocytes. The results showed that RNAi‐OPN3 reduced MMP in melanocytes (Fig. 4A). In RNAi‐OPN3 knockout of melanocytes, we also found that the protein expression level of cytochrome c was significantly higher than that of the control group (Fig. 4B). These data indicate that RNAi‐OPN3 induces mitochondrial damage in human melanocytes.

Cytochrome c interacts with monomeric APAF‐1 to promote conformational changes in the latter, resulting in oligomerization and recruitment of caspase 9 to form apoptotic bodies 32, 41. The associated caspase 9 is thereby activated, and this in turn cleaves and activates the executioner caspase 3 and caspase 7 39, 41. These then cleave key substrates in the cell to produce the cellular and biochemical events as observed in apoptosis. In the RNAi‐OPN3 experimental group, we also found that the protein expression levels of cleaved caspase 9, caspase 9, cleaved caspase 3, caspase 3, cleaved caspase 7 and caspase 7 were significantly higher than those of the control group (Fig. 4B). All of these results indicate that RNAi‐OPN3 reduces calcium influx and induces mitochondrial damage in human melanocytes, leading to apoptosis in cells. We conclude, therefore, that OPN3 is a key protein molecule that regulates the survival of melanocytes.

## Discussion

OPN3 is a G protein‐coupled membrane receptor with a wide range of distribution including human eyes, brain, testes, liver, kidneys and skin 11, 12, 19, 20, 21, 22. However, its functions are not completely understood. Recent data have shown that OPN3 is highly expressed in melanocytes and keratinocytes 24, 26. OPN3 may be a sensor in melanocytes responsible for blue‐induced hyperpigmentation 24. OPN3 also plays light‐independent roles in asthma and cell cycle modulation of hair follicle cells 23. Here, we provide evidence to support the notion that OPN3 is a key protein molecule for the survival of human epidermal melanocytes. We demonstrate that knockdown of OPN3 in melanocytes leads to apoptosis through a calcium‐dependent G protein‐coupled signaling and mitochondrial pathway.

It is well‐known that melanocytes are pigment‐producing cells of the skin in humans and other vertebrates 44, 45. Melanocytes originate from the neural crest with pluripotential cells that gradually become lineage specific during development 46. The cell survival, proliferation, migration and self‐renewal of melanocytes are regulated by various signaling molecules including MITF, c‐kit, snail/slug, sox10 and endothelins 2, 3, 4, 5. However, key protein molecules that regulate the survival of melanocytes remain unknown. In this study, we demonstrate that OPN3 is essential for the survival of melanocytes. When OPN3 gene was silenced, melanocytes underwent apoptosis (Fig. 2).

Apoptosis plays an important role in the development and maintenance of tissue homeostasis 32, 33. There are two major signal pathways (extrinsic and intrinsic) in apoptosis depending on the nature of the death‐inducing signals 32, 47. The extrinsic pathway is activated by the binding of death‐inducing ligands to cell‐surface death receptors 48. The intrinsic pathway, also known as the mitochondrial pathway, is initiated by cell‐intrinsic apoptotic stimuli include DNA damage, growth factor withdrawal and oxidative stress 49. The mitochondrial pathway is mainly regulated by Bcl‐2 family members, in which both MOMP and cytochrome c play a crucial role 40, 50. Exact mechanism for the apoptosis of melanocytes has not been clear now. Autoimmune factors are considered to one of the most influential pathogenic causes 51. Also, oxidative stress induces the apoptosis of melanocytes in a mitochondria‐dependent way, which mainly involves modulation of the mitochondrial‐dependent caspase activation and p38 MAPK pathway 52.

How can OPN3 knockdown lead to apoptosis? We sought to uncover the signaling pathway leading to this OPN3‐mediated phenomenon. In this study, we found that downregulation of OPN3 markedly reduces the intracellular calcium level and decreases the phosphorylation level of BAD (Fig. 3B). The reduced amount of phosphorylated BAD and elevation level of BAD altered MMP, which triggered activation of BAX and inhibition of BCL‐2 and raf‐1. Members of the B cell lymphoma 2 (BCL‐2) gene family have a central role in regulating programmed cell death by controlling proapoptotic and antiapoptotic intracellular signals 50, 53. These antiapoptotic proteins include Bcl‐2 and Bcl‐XL, while proapoptotic proteins include Bax, Bak, Bid and Bad 32, 54. Previous studies have shown that lowering the Bcl2/Bax ratio promotes mitochondrial outer membrane permeabilization (MOMP) 39. Since MOMP is primarily regulated through interactions between proapoptotic and antiapoptotic proteins of B cell lymphoma 2 (BCL‐2) family 40, MMP decreases following MOMP during apoptosis. We used JC‐1 staining method to detect MMP of RNAi‐OPN3 melanocytes and found that RNAi‐OPN3 can reduce MMP in melanocytes (Fig. 4A). As previously reported, mitochondrial depolarization results in the release of cytochrome c (Fig. 4B). In turn, cytochrome c binds to apoptosis protease‐activating factor 1 (APAF 1) and procaspase 9, generating an intracellular “apoptosome” that activates caspase 9 and cleaved caspase 9 (Fig. 4B). Caspase 9 then activates procaspase 3 and procaspase 7 (Fig. 4B), resulting in cell death (Fig. 2). Caspase 3 and caspase 7 are key mediators of the terminal events that drive apoptosis y 55. These results indicate that knockdown of OPN3 induces melanocyte apoptosis through the intrinsic death pathway or mitochondrial pathway.

In summary, our research focuses on the effects of the intrinsic function of OPN3 on the survival of human epidermal melanocytes in vitro. OPN3 knockdown triggers apoptosis in human epidermal melanocytes is *via* calcium‐dependent G protein‐coupled signaling and mitochondrial pathway (Fig. 4C). This is the first report to demonstrate that OPN3 controls the survival of human epidermal melanocytes by maintaining the antiapoptosis effect.
